# The impact of gender differences on the clinical characteristics of critically ill patients with venous thromboembolism: A retrospective, observational study

**DOI:** 10.1097/MD.0000000000038423

**Published:** 2024-06-14

**Authors:** Meng He, Jiuhang Ye, Weiwei Zheng, Peng Qiao, Haiyan Gu, Wenwen Qin, Xuehong He

**Affiliations:** a Department of Prehospital Emergency, Affiliated Hospital of Jining Medical University, Jining, Shandong, P. R. China; b Department of Critical Care Medicine, Affiliated Hospital of Jining Medical University, Jining, Shandong, P. R. China; c Department of Emergency Intensive Care Unit, Affiliated Hospital of Jining Medical University, Jining, Shandong, P. R. China; d Emergency Internal Medicine Ward, Affiliated Hospital of Jining Medical University, Jining, Shandong, P. R. China; e Department of Nursing, Affiliated Hospital of Jining Medical University, Jining, Shandong, P. R. China.

**Keywords:** clinical characteristics, gender differences, ICU, VTE

## Abstract

The aim of this study is to describe the general information, disease and treatment of venous thromboembolism (VTE) in critically ill patients, to explore the characteristics of severe patients with deep venous VTE and provide clinical reference data for the prevention and treatment of VTE in critically ill patients. This study carried out a retrospective study on the medical records of patients who were treated in the intensive care unit of Affiliated Hospital of Jining Medical College from 2020 to 2022. The general data, general conditions, drug use, past history, VTE prevention measures, post-VTE conditions, and Padua risk score of 297 patients with VTE during the period of hospitalization in intensive care unit (ICU) were classified and statistically analyzed. A total of 297 including 171 male and 126 male patient medical records were included in the analysis, we compared multiple clinical indicators between male and female patients. Compared to women, male patients have a higher acute physiology and chronic health evaluation II score(APACHE-II score) (*P* = .027), a higher state of consciousness (*P* = .003), a higher rate of smoking and drinking history (*P* < .001), a lower rate of heart failure (*P* = .003) and chronic depression (*P* = .013), and a higher rate of recommended operations for male patients after VTE (*P* = .031). The prothrombin time (PT) (*P* = .041) and activated partial thromboplastin time (APTT) (*P* = .040) of male patients were significantly higher than those of female patients, while triglyceride (*P* = .009) and total cholesterol (TC) (*P* = .001) were significantly lower than those of female patients. The difference in D-dimer (*P* = .739) was not significant. This study shows that male and female patients with VTE in the ICU have significant differences in general characteristics, general clinical conditions, history of past illness, thrombosis character, and examination items, this may be the reason for the different rates of VTE between male and female patients in the ICU.

## 1. Introduction

Venous thromboembolism (VTE) is a prevalent form of cardiovascular disease.^[1–3]^ While the exact pathogenesis of VTE remains incompletely understood,^[4]^ factors such as dietary type,^[5]^ diabetes,^[6]^ cancer,^[7,8]^ genetic factors,^[9]^ pregnancy,^[10]^ stroke,^[11]^ aging,^[12]^ and hormone level changes^[13,14]^ have been associated with its occurrence. Undiagnosed VTE poses a greater risk compared to cases with a definite diagnosis.^[15]^ Therefore, early diagnosis and prevention of VTE are crucial for improving patient outcomes. Individuals who have undergone surgery or recent hospitalization are at higher risk of developing VTE.^[16]^ Additionally, the risk of VTE is elevated in intensive care unit (ICU) patients.^[17–19]^ Exploring the risk factors and effective preventive measures for VTE in the ICU setting may contribute to better rehabilitation outcomes for these patients.^[20,21]^ Furthermore, men are more susceptible to VTE compared to women,^[22]^ and estrogen therapy has been shown to reduce the risk of VTE.^[23]^ Thus, we hypothesize that gender differences may impact VTE-related risk factors. In this retrospective study, we analyzed data from patients with VTE admitted to the intensive care unit(ICU) of Jining Medical College Affiliated Hospital between 2020 and 2022. The primary objective of this study is to investigate the influence of gender differences on the clinical characteristics of critically ill patients. Our findings aim to provide clinicians with valuable reference data for identifying patients at high risk of VTE and implementing appropriate prevention and treatment strategies.

## 2. Methods

This is a retrospective, data-only, observational study. The Medical Science Research Ethics Committee of the Affiliated Hospital of Jining Medical College approved this study (No. 2023-04-C026), individual consent was waived.

### 2.1. Study design

This study retrospectively collected and analyzed the medical records of patients with VTE in the ICU of Affiliated Hospital of Jining Medical College from January 2020 to October 2022. After excluding incomplete medical records, a total of 101 patient medical records were included in the study, using gender as the sole variable, conduct a significant difference analysis on the information in medical records.

### 2.2. Data collection

This study collected general information, clinical conditions, past medical history, VTE characteristics, and laboratory examination data from the medical records of the included patients. Conduct a significant difference analysis using gender as a variable for each item of information.

### 2.3. Statistical analysis

The data was statistically analyzed using the SPSS software (version 23.0; SPSS Inc., Chicago, IL). For metric data that did not conform to a normal distribution, the median (quartile) was used for representation. The Wilcoxon rank-sum test was employed for intergroup comparisons. Count data was represented by frequency and percentage, with intergroup comparisons conducted using the Chi-square test or corrected Chi-square test. A *P* value of <.05 was considered statistically significant.

### 2.4. Data availability

Anonymized data on which this article is based will be shared on reasonable request with any appropriately qualified investigator.

## 3. Result

### 3.1. General information of patients

Table 1 shows that there are significant differences in the marital status, education level, living environment, history of drinking and smoking of the patients included in this study. There is no significant difference in the Nutrition score, but the APACHE-II (Acute Physiology and Chronic Health Evaluation II) score of male patients is significantly higher than that of female patients.

**Table 1:** Patient general characteristics.

Characteristics	Male (n = 171)	Female (n = 126)	Z or χ^2^	*P* value
Age, average (range)	69 (58,77)	69 (60,79)	−0.764	.445
BMI (range)	23.3 (20.76,26.23)	22.97 (20,25.5)	−0.961	.336
Marital status			14.248	.001
Unmarried, n (%)	5 (2.92%)	0 (0%)	2.189	.139
Married, n (%)	145 (84.8%)	93 (73.81%)	5.5	.019
Divorce, n (%)	4 (2.34%)	2 (1.59%)	0.001	.97
Widowed, n (%)	17 (9.94%)	31 (24.6%)	11.509	.001
Level of education			57.198	.000
Illiteracy, n (%)	16 (9.36%)	57 (45.24%)	50.384	0
Junior high school and below, n (%)	111 (64.91%)	60 (47.62%)	8.882	.003
Senior middle school, n (%)	24 (14.04%)	7 (5.56%)	5.580	.018
Junior college education, n (%)	12 (7.02%)	2 (1.59%)	4.763	.029
Bachelor degree or above, n (%)	8 (4.68%)	0 (0%)	4.404	.036
Living environment			7.601	.006
Countryside, n (%)	109 (63.74%)	99 (78.57%)	7.601	.006
Downtown, n (%)	62 (36.26%)	27 (21.43%)	7.601	.006
Smoking history			87.095	.000
No smoking, n (%)	68 (39.77%)	117 (92.86%)	87.051	0
Smoke, n (%)	41 (23.98%)	3 (2.38%)	26.809	0
Quit smoking, n (%)	62 (36.26%)	6 (4.76%)	40.764	0
History of Drinking			86.623	.000
Drink, n (%)	72 (42.11%)	119 (94.44%)	86.584	0
No drinking, n (%)	49 (28.65%)	4 (3.17%)	32.127	0
Quit drinking, n (%)	50 (29.24%)	3 (2.38%)	35.697	0
Nutrition score	3 (3,4)	3 (3,4)	−0.366	.714
Acute Physiology and Chronic Health Evaluation II (APACHE-II) score, average (range)	18 (14,24)	17 (11,21.25)	−2.208	.027

Continuous variables are represented by median (range). APACHE- II score = Acute Physiology and Chronic Health Evaluation II score, BMI = body mass index.

### 3.2. General clinical conditions of patients

Table 2 shows there are significant differences in the state of consciousness, oxygen intake method, diet frequency, and lower limb edema between male and female patients with VTE in the ICU.

**Table 2:** General clinical conditions.

Status	Male (n = 171)	Female (n = 126)	Z or χ^2^	*P* value
State of consciousness			17.990	.003
Calm, n (%)	57 (33.33%)	59 (46.83%)	5.548	.019
Sober, n (%)	4 (2.34%)	8 (6.35%)	3.009	.083
Lethargy, n (%)	30 (17.54%)	14 (11.11%)	2.379	.123
Confusion, n (%)	11 (6.43%)	13 (10.32%)	1.474	.225
Lethargy, n (%)	61 (35.67%)	32 (25.4%)	3.561	.059
Deliration, n (%)	8 (4.68%)	0 (0%)	4.404	.036
Oxygen inhalation method			−3.646	.000
None, n (%)	0	0	10.241	.001
Nasal catheter, n (%)	67 (39.18%)	73 (57.94%)	0.032	.858
Oxygen mask n, (%)	10 (5.85%)	8 (6.35%)	0	1
Anesthesia mask, n (%)	1 (0.58%)	1 (0.79%)	0	1
Noninvasive ventilator, n (%)	2 (1.17%)	1 (0.79%)	0.128	.721
Tracheal cannula, n (%)	27 (15.78%)	18 (14.29%)	2.652	.103
Tracheotomy, n (%)	11 (6.43%)	3 (2.38%)	7.960	.005
Artificial airway connected to ventilator, n (%)	53 (30.99%)	21 (16.67%)	2.128	.145
Oxygen therapy time (hours)	19 (6216)	14.5 (5,96)	−1.168	.243
Diet frequency			8.843	.031
Fasting, n (%)	92 (53.8%)	57 (45.24%)	2.128	.145
3 times per day, n (%)	22 (12.87%)	30 (23.81%)	6.016	.014
More than 3 times a day, n (%)	5 (2.92%)	8 (6.35%)	2.033	.154
Continuous enteral nutrition, n (%)	52 (30.41%)	31 (24.6%)	1.215	.27
Deep vein/PICC catheterization, n (%)	37 (21.64%)	19 (15.08%)	4.157	.090
Lower limb indwelling vein catheterization/indwelling needle, n (%)	2 (1.17%)	1 (0.79%)	0 (0.0)	1.000
The limb at the side of the venous catheter is swollen, and the peripheral diameter of the limb is ≥ 1cm n (%)	21 (12.28%)	11 (8.73%)	0.951	.329
Varicose veins of lower limbs, n (%)	1 (0.58%)	1 (0.79%)	0.000	1.000
Lower limb edema, n (%)	8 (4.68%)	13 (10.32%)	3.511	.061

### 3.3. History of past illness

Table 3 shows that female patients with VTE have a significantly higher probability of Heart failure and Chronic nephrosis than male patients, there was no significant difference in medication history after admission.

**Table 3:** History of past illness and recent medication history.

Disease	Male (n = 171)	Female (n = 126)	Z or χ^2^	*P* value
Coronary disease, n (%)	48 (28.07%)	43 (34.13%)	1.252	.263
Hypertension, n (%)	84 (49.12%)	50 (39.68%)	2.426	.119
Diabetes, n (%)	34 (19.88%)	32 (25.4%)	1.276	.259
Hyperlipidemia, n (%)	2 (1.17%)	5 (3.97%)	1.403	.236
Stroke, n (%)	33 (19.3%)	23 (18.25%)	0.052	.820
Atrial fibrillation, n (%)	14 (8.19%)	13 (10.32%)	0.398	.528
Heart failure, n (%)	1 (0.58%)	10 (7.94%)	9.121	.003
Pulmonary disease, n (%)	45 (26.32%)	34 (26.98%)	0.017	.897
Chronic nephrosis, n (%)	4 (2.34%)	11 (8.73%)	6.179	.013
Blood system disease, n (%)	0 (0%)	2 (1.59%)		.179
Digestive system disease, n (%)	11 (6.43%)	14 (11.11%)	2.060	.151
Autoimmune diseases	1 (0.58%)	2 (1.59%)	0.071	.790
Varicose veins	3 (1.75%)	1 (0.79%)	0.040	.841
Thrombophlebitis	4 (2.34%)	3 (2.38%)	0.000	1.000
Malignant tumors	11 (6.43%)	8 (6.35%)	0.001	.977
Acute inflammatory lesions	160 (93.57%)	117 (92.86%)	0.058	.809
Taking contraceptive pills within 3 months	0 (0%)	1 (0.79%)	0.204	.878
Application of antibiotics after hospitalization	79 (46.2%)	60 (47.62%)	0.724	.946
Application of dehydrating diuretic drugs after hospitalization	60 (35.09%)	41 (32.54%)	0.923	.833
Application of anticoagulants after hospitalization	14 (8.19%)	14 (11.11%)	0.726	.394
Application of thrombolytic drugs after hospitalization	4 (2.34%)	2 (1.59%)	0.001	.970
Application of hormone drugs after hospitalization	1 (0.58%)	5 (3.97%)	2.660	.103

### 3.4. Thrombosis character, nursing measures and VTE consultation opinions

Table 4 shows the ultrasound results of VTE patients show significant differences in the distribution of deep vein thrombosis between male and female VTE patients. After the occurrence of VTE, there is a significant difference in the medication situation between male and female patients. In nursing interventions, the probability of female patients wearing stretch socks and receiving limb pressure therapy is higher than that of males. In VTE consultation opinions, the probability of recommending surgery for male patients is significantly higher than that of females.

**Table 4:** VTE location, positive signs, and nursing measures.

Status	Male (n = 171)	Female (n = 126)	Z or χ^2^	*P* value
Limb distribution			8.355	.025
Left lower limb	39 (22.81%)	37 (29.37%)	1.639	.201
Right lower limb	33 (19.3%)	35 (27.78%)	2.955	.086
Both lower limbs	99 (57.89%)	54 (42.85%)	6.568	.01
Location of thrombus			1.400	.743
Peripheral type	130 (76.02%)	103 (81.75%)	1.405	.236
Central type	3 (1.75%)	2 (1.59%)	0	1
Mixed type	35 (20.47%)	20 (15.87%)	1.015	.314
VTE positive signs				
Pain/tenderness	1 (0.58%)	1 (0.79%)	1.152	.562
Limb swelling	7 (4.09%)	9 (7.14%)	1.323	.250
Low limb temperature	4 (2.34%)	1 (0.79%)	0.321	.571
Abnormal limb color	2 (1.17%)	1 (0.79%)	0.000	1.000
Lower limb asymmetry circumferential diameter difference ≥ 1cm	81 (47.37%)	54 (42.86%)	0.595	.440
Positive gastrocnemius traction test	1 (0.58%)	0 (0%)	0.000	1.000
Loss of pulsation of dorsal foot artery	1 (0.58%)	2 (1.59%)	0.068	.794
Medication			13.344	.012
None	121 (70.76%)	84 (66.67%)	0.569	.451
low-molecular-weight heparin sodium	7 (4.09%)	3 (2.38%)	0.234	.629
low-molecular-weight heparin calcium	0 (0%)	6 (4.76%)	6.079	.014
Enoxaparin	3 (1.75%)	4 (3.17%)	0.168	.681
Nadroparin	40 (23.39%)	26 (20.63%)	0.319	.572
Rivaroxaban	0 (0%)	3 (2.38%)	2.076	.15
Nursing intervention				
Knowledge education on VTE	169 (98.83%)	124 (98.41%)	0.000	1.000
No getting out of bed	169 (98.83%)	126 (100%)	0.250	.617
Raise the affected limb	2 (1.17%)	0 (0%)	0.250	.617
Non-drug prevention				
Avoid lower limb venous puncture and infusion	171 (100%)	124 (98.41%)	0.875	.350
Quit smoking and drinking	161 (94.15%)	113 (89.68%)	2.028	.154
Low salt and low fat diet	154 (90.06%)	109 (86.51%)	0.902	.342
Moderate fluid replacement	157 (91.81%)	111 (88.1%)	1.138	.286
Wear stretch socks	28 (16.37%)	38 (30.16%)	7.975	.005
Ankle pump movement	163 (95.32%)	122 (96.83%)	0.423	.515
Limb pressure therapy	53 (30.99%)	54 (42.86%)	4.430	.035
VTE consultation opinions				
None	35 (20.47%)	21 (16.67%)	0.685	.408
Medication treatment	88 (51.46%)	53 (42.06%)	2.570	.109
Nursing measures	45 (26.32%)	31 (24.6%)	0.112	.738
Operation	45 (26.32%)	20 (15.87%)	4.628	.031

VTE = venous thromboembolism.

### 3.5. Examination items

Table 5 shows the thrombosis character and nursing measures of the patients. Prothrombin time (PT), activated partial thromboplastin time(APTT) data showed that the blood clotting level of male patients is higher than that of females, the C-reactive protein level of male patients is higher than that of female patients, the triglyceride and total cholesterol (TC) levels of females are higher than those of males, the creatinine and urea levels of male patients are higher than those of females, and the PaCO_2_ and K^+^ levels of male patients are higher than those of female patients.

**Table 5:** Examination items.

Examination items	Male (n = 171)	Female (n = 126)	Z or χ^2^	*P* value
Coagulation routine				
PT	13.5 (12.4, 14.7)	12.9 (12, 14.43)	−2.046	.041
APTT	29.7 (27.4, 33.7)	29.15 (25.53, 32)	−2.049	.040
TT	14.9 (13.5, 16.9)	15.6 (13.78, 16.8)	−0.958	.338
Plasma fibrinogen (g/L)	3.8 (2.9, 4.7)	3.45 (2.6, 4.57)	−1.289	.197
Haemocytes				
Red blood cell (10^9^/L)	3.64 (3.09, 4.44)	3.76 (3.21, 4.19)	−0.392	.695
White blood cell (10^9^/L)	9.64 (7.67, 13.8)	10.59 (7.72, 14.58)	−0.969	.332
Platelet (10^9^/L)	208 (151, 272)	214 (147.75, 322)	−0.804	.421
Blood biochemical analysis				
D-dimer	2.35 (1.08, 9.66)	3.34 (1.23, 6.73)	−0.333	.739
Brain natriuretic peptide	165.380 (57.00, 542.485)	141 (67, 2.920)	−0.0548	.956
C-reactive protein	42.36 (12.97, 88.21)	24.64 (4.71, 72.66)	−2.147	.032
Blood sugar	7.3 (5.9, 9.8)	8.15 (6.39, 10.8)	−1.889	.059
Blood fat				
TG	0.885 (0.59, 1.293)	1.075 (0.738, 1.493)	−2.631	.009
TC	3.18 (2.48, 3.94)	3.73 (2.98, 4.32)	−3.214	.001
HDL	0.96 (0.79, 1.22)	1.02 (0.81, 1.29)	−0.990	.322
LDL	1.96 (1.39, 2.63)	2.21 (1.57, 2.69)	−1.687	.092
Renal function				
Creatinine	68.1 (54.3, 92.6)	55 (40.65, 72.2)	−4.166	.000
Urea	7 (4.8, 11.3)	6.3 (3.9, 8.83)	−2.819	.005
Urea acid	222 (154, 314)	207.5 (131, 311.5)	−1.336	.182
β2 Microglobulin	2.36 (1.7, 3.92)	2.4 (1.58, 4.24)	−0.433	.665
Cysteine protease inhibitor	1.19 (0.89, 1.64)	1.11 (0.86, 1.6)	−0.804	.422
Blood gas analysis				
PH	7.43 (7.37, 7.48)	7.45 (7.4, 7.49)	−1.891	.059
PaO_2_	104 (71, 152)	102.5 (77.75, 145.75)	−0.263	.792
PaCO_2_	37 (31, 45)	35 (31, 40)	−2.122	.034
HCO_3_^-^	25.1 (22, 28.4)	24.4 (21.63, 28.33)	−0.041	.967
BE	1.4 (−1.2, 4.23)	1.25 (−2.4, 4.83)	−0.102	.918
K^+^	4.1 (3.7, 4.5)	3.9 (3.4, 4.3)	−2.593	.010
Na^+^	139 (134, 142)	138 (133, 141)	−1.322	.186
Lactic acid	1.3 (1, 2)	1.3 (0.98, 1.9)	−0.123	.902

APTT = activated partial thromboplastin time(s), HDL = high-density lipoprotein cholesterol, LDL = low density lipoprotein cholesterol, PT = prothrombin time(s), TC = Total cholesterol, TG = triglyceride, TT = thrombin time(s).

## 4. Discussion

VTE is a significant complication acquired in hospitals.^[24]^ Therefore, identifying patients at high risk of VTE is crucial for targeted prevention.^[25]^ In this study, there were more males (35.67%) and females (4.68%) who developed VTE in ICU patients, indicating that males who developed VTE may have a higher risk of VTE. There is also a significant difference between the oxygen intake method and the frequency of diet. Considering that male patients with VTE in the ICU have a higher APACHE- II score (*P* = .027), we speculate that male patients with poorer status in the ICU are more likely to develop VTE compared to females. Reduced APTT is associated with increased risk of VTE occurrence,^[26,27]^ the data of this study also shows that the PT (*P* = .041), APTT (*P* = .040) is significantly higher than that of female patients, indicating that the coagulation levels of these male patients are significantly higher than those of female patients, may be related to the use of anticoagulants.^[28,29]^ The clinical use of D-dimer testing in anticoagulated patients is very limited,^[30]^ but D-dimer enhances thrombus risk in cancer patients,^[31]^ blood biochemical analysis data showed that female patients had higher but not significant D-dimer than male patients (*P* = .739), and C-reactive protein was significantly less than male patients (*P* = .032). Creatinine and urea level can serve as an indicator of renal function,^[32]^ and an increase in blood urea nitrogen/creativity ratio may be related to an increased risk of VTE,^[33]^ the data of this study showed that male patients had higher creatinine(*P* < .001) and urea (*P* < .005) level than female patients, data from blood gas analysis showed that PaO_2_ (*P* = .034) and K^+^(*P* = .010) was significantly higher in male patients than in female patients, considering the preventive effect of non-vitamin K antagonist oral anticoagulants on VTE,^[34,35]^ we speculate that higher blood potassium levels in males are also one of the reasons for their higher incidence of VTE.

Some patients in the ICU exhibit hypercoagulability upon admission, making them primary subjects for VTE prevention.^[36]^ To mitigate the risk of VTE and its associated adverse effects, it is necessary to adopt appropriate care,^[37]^ pharmacological prevention for high-risk patients,^[38]^ and mechanical prevention,^[39]^ all of which are effective strategies. In addition to these prophylactic measures, therapeutic anticoagulation remains a fundamental strategy for treating VTE.^[40]^ Studies suggest that continuous anticoagulation in patients with VTE yields better clinical outcomes,^[41]^ low-molecular-weight heparins (LMWHs) have been used extensively to prevent and treat VTE,^[42,43]^ however, in the context of certain drugs and liver diseases, LMWHs may increase the risk of thromboembolism or overtreatment^[44]^ and potentially cause major bleeding.^[45]^ Consequently, numerous studies are investigating alternative preventive and therapeutic modalities for VTE.^[46–48]^

The risk of developing VTE decreases with increasing body mass index (BMI).^[49]^ Moreover, men appear to have a higher risk of developing VTE than women.^[50]^ Data from this study revealed that 57.6% of the patients were men, who tended to have a higher BMI than women, the present study is consistent with previous research. A genetic predisposition to smoking is associated with increased odds of VTE formation.^[51]^ Former study suggest that alcohol consumption was not associated with the risk of pulmonary embolism^[52]^ or VTE,^[53]^ this study show that the proportion of female patients who smoke (*P* < .001) and drink (*P* < .001) is significantly lower than that of male patients, indicating a possible correlation between smoking and alcohol consumption among male patients in the ICU and the occurrence of VTE.

Studies have shown a possible association between neoplasm and VTE.^[54,55]^ The data from this study shows that there is no significant difference in the proportion of malignant tumors between male and female patients with VTE in the ICU (*P* = .977). Previous studies have shown that patients with chronic kidney disease have an increased risk of developing VTE.^[56]^ The data from this study shows that female patients with VTE in the ICU have a significantly higher proportion of heart failure (*P* = .003) and chronic nephrosis (*P* = .013).

Thrombocytosis and a history of thrombosis are risk factors for the development of thrombotic events,^[57]^ and there was no significant difference in platelet levels between male and female patients in this study (*P* = .7873). Circulating metabolites may influence the risk of VTE,^[58]^ low density lipoprotein cholesterol, high-density lipoprotein cholesterol, triglyceride considered no causal relationship between them and VTE was found.^[59]^ The blood fat test in this study showed triglyceride (*P* = .009) and TC (*P* = .001) were significantly higher than those in male patients, indicating that blood lipid levels may affect the occurrence of VTE.

Ischemic brain injury causes increased levels of interleukin-17,^[60]^ inflammation may be one of the causes of VTE,^[61]^ and the detection of inflammation-related factors with gender difference as a variable has the potential to be a subject worth exploring. New crown pneumonia and hypercoagulability of blood are associated,^[62–64]^ the absence of statistical analysis of this potential contributing factor in this context is a weakness of this study.

The relevant research on VTE provides a basis for preventing and reducing its clinical harm, but the impact of gender differences on ICU patients with VTE is still unclear. This study retrospectively studied the clinical characteristics of male and female ICU patients with VTE, compared and analyzed the impact of gender on ICU patients with VTE, and provided reference for the prevention of VTE in ICU patients of different genders in clinical practice. Due to the limited ability of the research team, the number of medical records included in this study is relatively small, further multicenter, prospective, non-randomized controlled studies are needed.

## 5. Conclusion

This study shows that male and female patients with VTE in the ICU have significant differences in general characteristics, general clinical conditions, history of past illness, thrombosis character, and examination items, suggesting that these indicators may serve as potential targets for preventing VTE in patients in the ICU.

## Author contributions

**Conceptualization:** Meng He, Xuehong He.

**Data curation:** Meng He, Jiuhang Ye, Weiwei Zheng, Peng Qiao, Haiyan Gu, Wenwen Qin.

**Formal analysis:** Jiuhang Ye, Weiwei Zheng, Peng Qiao, Haiyan Gu, Wenwen Qin.

**Investigation:** Meng He, Jiuhang Ye, Weiwei Zheng, Xuehong He.

**Methodology:** Meng He, Jiuhang Ye, Weiwei Zheng, Peng Qiao, Haiyan Gu, Wenwen Qin.

**Project administration:** Xuehong He.

**Resources:** Xuehong He.

**Writing – original draft:** Meng He, Xuehong He.

**Writing – review & editing:** Xuehong He.
